# Oral *Prevotella* Species and Their Connection to Events of Clinical Relevance in Gastrointestinal and Respiratory Tracts

**DOI:** 10.3389/fmicb.2021.798763

**Published:** 2022-01-06

**Authors:** Eija Könönen, Ulvi K. Gursoy

**Affiliations:** Institute of Dentistry, University of Turku, Turku, Finland

**Keywords:** anaerobic bacteria, commensalism, dysbiosis, inflammation, microbiology, *Prevotella*, systemic disease, taxonomy

## Abstract

*Prevotella* is recognized as one of the core anaerobic genera in the oral microbiome. In addition, members of this genus belong to microbial communities of the gastrointestinal and respiratory tracts. Several novel *Prevotella* species, most of them of oral origin, have been described, but limited knowledge is still available of their clinical relevance. *Prevotella melaninogenica* is among the anaerobic commensals on oral mucosae from early months of life onward, and other early colonizing *Prevotella* species in the oral cavity include *Prevotella nigrescens* and *Prevotella pallens*. Oral *Prevotella* species get constant access to the gastrointestinal tract via saliva swallowing and to lower airways via microaspiration. At these extra-oral sites, they play a role as commensals but also as potentially harmful agents on mucosal surfaces. The aim of this narrative review is to give an updated overview on the involvement of oral *Prevotella* species in gastrointestinal and respiratory health and disease.

## Introduction

Anaerobic bacteria constitute a significant part of oral microbial communities. In the oral cavity, Bacteroidetes is one of the major phyla and *Prevotella* its largest genus (Dewhirst et al., 2010). This genus, which has expanded significantly during the past decades, consists of gram-negative, strictly anaerobic, mainly short rod-shaped bacteria. In healthy adults, detection rates of *Prevotella* organisms are high in saliva and dental plaque (Keijser et al., 2008; Xu et al., 2015). In saliva, richness of the diversity within this genus is especially high (Keijser et al., 2008). At the species level, however, the majority of data currently available deals with *Prevotella intermedia* and/or *Prevotella nigrescens* due to their clinical relevance in oral pathologies but also ignorance of commensals. A study looking for intraoral distribution of bacterial species in 225 systemically healthy individuals showed *Prevotella melaninogenica* in high proportions in saliva and at the dorsum and lateral sites of the tongue (Mager et al., 2003).

**FIGURE 1:**
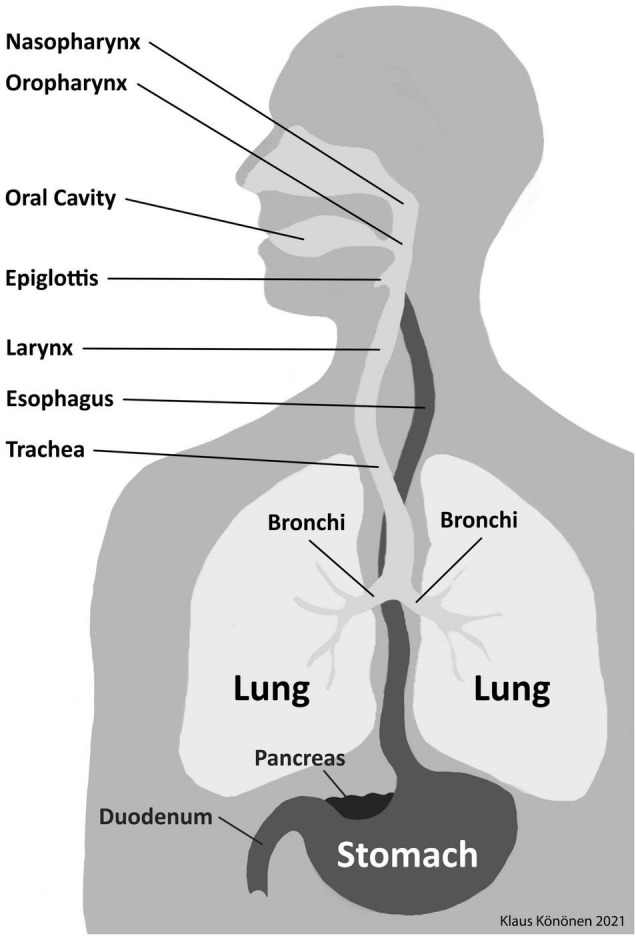
The anatomy and potential habitats in the aerodigestive tract in humans.

In the context of this review, human habitats exposed to oral bacteria to be colonized outside the oral cavity are presented in Figure 1. There are obvious differences in the microbial communities between body habitats and between individual metabolic niches (Costello et al., 2009; Segata et al., 2012). At the genus level, *Prevotella* is frequent and widespread all over on the surfaces of the human body; however, a species-level identification methodology is necessary for revealing whether same species colonize throughout the gastrointestinal tract and whether their relative abundance varies among the habitats as well as inter-individually (Segata et al., 2012; Schmidt et al., 2019). *Prevotella* strains present in saliva are of particular interest. Due to constant saliva swallowing of approximately 1.5 L daily (Humphrey and Williamson, 2001), this oral fluid is the most plausible vehicle for oral microorganisms and their biologically active components to be translocated to other parts of the digestive tract (Schmidt et al., 2019). Oral bacteria surviving to pass acidic circumstances of the stomach get access to the small intestine and colon, where they can interfere with intestinal bacteria (Schmidt et al., 2019; Kitamoto et al., 2020). At the genus level, *Prevotella* is a common colonizer of these distant habitats. However, at the species level, distinct bacterial populations occupy the oral and intestinal microenvironments. Also immune cells involved in chronic inflammatory processes in the mouth end up via saliva to the gut, sometimes resulting in pathological consequences (Byrd and Gulati, 2021).

In the respiratory tract, potential routes for bacterial translocation from the oral cavity include (micro)aspiration, in particular, and hematogenous spread. Indeed, recent studies have shaken our views on the oral source of bacteria for the composition of microbial communities of the lower respiratory tract (Bassis et al., 2015; Dickson et al., 2016). Currently, *Prevotella* is considered one of the major genera colonizing mucosal surfaces of the aerodigestive tract, including the lungs, which were long seen as a sterile site in the human body.

In this narrative review, the purpose is to give an updated overview on the presence of oral *Prevotella* species as members of the gastrointestinal and respiratory microbiota and on their involvement in diseases at these sites above the waistline.

## Taxonomical Overview of Oral *Prevotella*

In 1990, the genus *Prevotella* was described by reclassifying a group of moderately saccharolytic and bile-susceptible *Bacteroides* species as members of this novel genus (Shah and Collins, 1990). The name *Prevotella* came after A. R. Prévot, who was a French microbiologist with pioneering expertise in anaerobic bacteriology. Most of these reclassified *Prevotella* organisms were recovered from the oral cavity of humans, *P. melaninogenica* being the type species of the genus. At that time, pigment production of colonies on blood agar was seen as an important feature to divide the organisms into pigmented and non-pigmented *Prevotella* species (Jousimies-Somer et al., 2002). In addition to *P. melaninogenica*, the pigmented group included *Prevotella loescheii*, *Prevotella denticola* (part of strains), *P. intermedia*, and *Prevotella corporis*, their color intensity varying from light brown to black. Some years later, the pigmented group within *Prevotella* was expanded by two phylogenetically closely related species, *P. nigrescens* (Shah and Gharbia, 1992) and *Prevotella pallens* (Könönen et al., 1998). During the 1990s, there was a notable research interest in black-pigmented gram-negative anaerobes, including *Porphyromonas* and *Prevotella* species, due to their link to various pathologies in humans (Finegold et al., 1993).

After the creation of the genus *Prevotella* and reclassification of moderately saccharolytic *Bacteroides* as *Prevotella* species by Shah and Collins (1990), there has been a great expansion of the genus with novel species, most of them of oral origin. Table 1 presents the validly published *Prevotella* species that have been primarily isolated from the oral cavity: 12 species formerly classified as *Bacteroides* and 18 novel *Prevotella* species described after 1990. On the other hand, a couple of reclassifications were made; while the former *Mitsuokella dentalis* is now *Prevotella dentalis* (Willems and Collins, 1995), *Prevotella tannerae* was removed to a novel, closely related genus *Alloprevotella*, also including a novel oral species, *Alloprevotella rava* (Downes et al., 2013). Two species with a distant phylogeny, *Prevotella heparinolytica* and *Prevotella zoogleoformans*, remain “on the waiting list” to be removed from the genus *Prevotella* (Dewhirst et al., 2010).

**TABLE 1:** Validly published *Prevotella* and *Alloprevotella* species with the primary isolation from the human oral cavity.

Year/Reference	Former *Bacteroides* sp. reclassified as *Prevotella* sp.	Year/References	Novel *Prevotella* and *Alloprevotella* spp.	Comments
Shah and Collins, 1990	*P. melaninogenica* (type species)	Shah and Gharbia, 1992	*P. nigrescens*	
	*P. buccae*	Moore et al., 1994	*P. enoeca* *P. tannerae*	
	*P. buccalis*	Willems and Collins, 1995	*P. dentalis*	*Mitsuokella dentalis*
	*P. denticola*	Könönen et al., 1998	*P. pallens*	
	*P. heparinolytica*	Sakamoto et al., 2004	*P. salivae* *P. shahii*	
	*P. intermedia*	Sakamoto et al., 2005a	*P. multiformis*	
	*P. loescheii*	Sakamoto et al., 2005b	*P. multisaccharivorax*	
	*P. oralis*	Downes et al., 2005	*P. baroniae* *P. marshii*	
	*P. oris*	Downes et al., 2007	*P. maculosa*	
	*P. oulorum*	Downes et al., 2008	*P. histicola*	
	*P. veroralis*	Downes et al., 2009	*P. micans*	
	*P. zoogleoformans*	Sakamoto et al., 2010	*P. aurantiaca*	
		Downes et al., 2010	*P. saccharolytica*	
		Downes and Wade, 2011	*P. fusca* *P. scopos*	
		Downes et al., 2013	*A. rava* *A. tannerae*	*Alloprevotella* gen. nov., reclassification of *P. tannerae*

In addition to the above-mentioned species principally isolated from the oral cavity, several novel *Prevotella* species have been described based on a few strains, or even a single strain, from various clinical specimens. Origins of these strains were as follows: two *Prevotella amnii* strains from amniotic fluid (Lawson et al., 2008), eight *Prevotella bergensis* strains from skin and soft tissue infections (Downes et al., 2006), one *Prevotella brunnea* strain from a wound at foot (Buhl et al., 2019), one *Prevotella colorans* strain from a wound (Buhl et al., 2016), three *Prevotella jejuni* strains from the jejunum of a celiac child (Hedberg et al., 2013), three *Prevotella nanceiensis* strains from bronchial fluid, lung abscess, or blood (Alauzet et al., 2007), one *Prevotella pleuritidis* strain from pleural fluid (Sakamoto et al., 2007), one *Prevotella timonensis* strain from breast abscess (Glazunova et al., 2007), and one *Prevotella vespertina* strain from an abscess locating at the upper respiratory tract (Buhl and Marschal, 2020). With this limited information given, the knowledge of their preferred habitat remains open.

Three former *Bacteroides* species, reclassified as *Prevotella bivia*, *Prevotella corporis*, and *Prevotella disiens* (Shah and Collins, 1990) come mainly from the human urogenital tract but there are also occasional recoveries from the mouth. In the current literature, several novel *Prevotella* species from stool specimens have been described as colonizers of the colon. Of those, *Prevotella copri* has drawn remarkable attention due to its potential beneficial effects on human well-being (Tett et al., 2021), although its connections to chronic inflammatory conditions of the gut have been recognized, too (Ley, 2016).

Besides biochemical and physiological testing, methods for *Prevotella* classification have traditionally included DNA–DNA hybridization, measuring of G + C content, and multilocus enzyme electrophoresis, however, classification gradually changes toward genomic methods. Full-sequencing of the 16S rRNA gene is a routine, but also comparison to whole-genome sequence databases is available and has been used in recent *Prevotella* descriptions (Buhl et al., 2016, 2019; Buhl and Marschal, 2020). Tett et al. (2021) recently described genomic characteristics of 25 named human *Prevotella* species, among those 17 species with genomes of multiple oral isolates in the analysis. Varying genome lengths between 2.37–4.26 Mb and G + C contents between 36.4–56.1% as well as the high number of core genes speak for the wide diversity within this genus.

## *Prevotella* in Bacterial Communities of the Digestive Tract

### *Prevotella* as Members of the Core Microbiota in the Gastrointestinal Tract

Concerning the alimentary tract from the oropharynx downward above the waistline, it was for long considered of being without a resident microbiota until advanced molecular methods allowed to challenging these earlier beliefs, which were based on negative cultures by routine microbiology methods from the esophagus and stomach (Pei et al., 2004; Bik et al., 2006). Pei et al. (2004) tested their hypotheses on the presence of indigenous bacteria on esophageal mucosa and on their fastidious nature, i.e., mainly being uncultivable. Biopsy specimens from the distal esophagus of four healthy individuals were examined with 16S rDNA sequencing-based techniques, and the results showed Bacteroidetes as the second most common phylum and *Prevotella* as the second most common genus after *Streptococcus*. *P. pallens* was among the 14 bacterial taxa found in all individuals, while many other named *Prevotella* species were also recovered: *Prevotella veroralis* from three individuals, *P. intermedia* and *P. melaninogenica* from two individuals, and *P. denticola*, *P. nigrescens*, and *Prevotella oris* each from one individual. In addition, several not-yet-named *Prevotella* clones were among the findings (Pei et al., 2004). To increase the understanding of the esophageal microbiome and its function in the host, Deshpande et al. (2018) studied the esophageal microbiome of over 100 individuals, with special emphasis on age, gender, proton pump inhibitor use, host genetics, and development of esophageal disease. They demonstrated that the esophageal microbiome clusters into functionally distinct bacterial community types. Cluster 3 was dominated by *P. melaninogenica* and *P. pallens*, and cluster 2 by mitis group streptococci, while cluster 1 was an intermediate type with respect to abundances of *Streptococcus* and *Prevotella*, indicating their ratio of being a significant factor in defining esophageal community types (Deshpande et al., 2018). It was shown that various, distinct pathways are increased in each community type. Age was observed to affect relative abundances within these two major genera; age positively correlated with *Streptococcus parasanguinis* (but not with mitis group streptococci), whereas age had an inverse correlation with *P. melaninogenica* (but not with *P. pallens*). Interestingly, there was a co-exclusion relationship between mitis group streptococci and *Prevotella* species across all esophageal disease stages (Deshpande et al., 2018).

Bik et al. (2006) collected gastric biopsy specimens from 23 adults, suffering from symptomatic upper gastrointestinal disease and being positive or negative for *Helicobacter pylori*. Advanced sensitive methods were used to reveal the bacterial composition of the gastric microbiota and the impact of *H. pylori* on its composition. There was a much larger bacterial diversity in gastric biopsies than was expected, but the sequence collection was also different from those of the mouth and esophagus presented in other studies. Interestingly, especially Bacteroidetes phylotypes were often absent from *H. pylori*-positive individuals (Bik et al., 2006). However, several recognized oral *Prevotella* species (and several *Prevotella* clones) were detected in individuals who were *H. pylori*-negative by conventional methods. Among the findings were, in descending order in their abundance, *P. melaninogenica* and *P. pallens*, in particular, and few *P. oris*, *P. intermedia*, *P. nigrescens*, *Prevotella oralis*, *Prevotella oulorum*, and *P. denticola*. It was emphasized that bacterial DNA may not indicate the presence of resident bacteria but, instead, could reflect the presence of bacterial cell remnants or transient flow of these bacteria in the specimens (Bik et al., 2006). Recently, it was confirmed that *H. pylori*, if present, dominates the bacterial community composition of the stomach, lowering the diversity and evenness of phylotypes (Schulz et al., 2018). Similarly to the study by Bik et al. (2006), two genera, *Streptococcus* and *Prevotella*, proved to dominate in bacterial communities of the upper gastrointestinal tract in *H. pylori*-negative individuals. By analyzing saliva, and stomach and duodenal aspirates and biopsies from a cohort of 24 individuals with chronic gastritis by high-throughput sequencing, Schulz et al. (2018) identified the oral cavity (saliva) of being the main source of active bacteria for the gastric microbiota, indicating a continuous migration of oral bacteria through the upper gastrointestinal tract. In individuals without *H. pylori*, no difference was seen in relative abundance of *Prevotella* and *Alloprevotella* between saliva and stomach aspirates, while reduced abundance for both genera was detected between the stomach and duodenum. In general, no significant difference at the genus level was observed in oral communities between individuals with or without *H. pylori*, but three phylotypes, among those *P. oris*, had significantly higher abundance in individuals without *H. pylori* (Schulz et al., 2018). Each individual harbored own specific bacterial communities throughout the upper gastrointestinal tract.

Saliva has been suggested as a key driver for the composition of bacterial communities in various habitats of the upper digestive tract, and as another key driver, a shared epithelial lining of their mucosae (Segata et al., 2012). Vasapolli et al. (2019) looked thoroughly for bacterial community changes in 21 healthy individuals throughout their gastrointestinal tract, including a wide selection of specimens: saliva, stomach (antrum and corpus), duodenum, terminal ileum, ascending and descending colon, and feces. They demonstrated that bacterial communities detected in samples from saliva, stomach, and duodenum formed 1 cluster and those from the lower gastrointestinal tract another cluster, while fecal communities differed from mucosa-associated findings of the gut (Vasapolli et al., 2019). The highest phylotype richness, including the genus *Prevotella*, was found in saliva, staying rather steady across the upper body sites, whereas in samples from lower parts of the body, a significantly reduced phylotype richness was observed. Among the phylum Bacteroidetes, abundance of *Prevotella* organisms present in saliva gradually decreased downward up to the duodenum, the decrease being drastic thereafter, whereas an opposite occurred to abundance of *Bacteroides* organisms (Vasapolli et al., 2019). In line were the results of a thorough culture-based study, performed in a homologous group of beagle dogs (Mentula et al., 2005), showing clear differences between jejunal and fecal samples and where a unique bacterial composition for each dog was found in small-intestinal fluid with a few species only at a time and fluctuating counts. Smoking seems to be a lifestyle factor affecting the mucosa-associated microbiota of the duodenum; in current smokers, a significantly reduced abundance of the phylum Bacteroidetes and the genus *Prevotella* was observed, and this reduction was only partially restored after quitting of smoking (Shanahan et al., 2018). *P. nanceiensis* was suggested to be a discriminatory species for previous smokers.

A few *Prevotella* species isolated from feces are considered having their habitat in the intestine. According to the current knowledge, especially *P. copri* is both a prevalent and abundant organism in the gut [see an excellent review by Tett et al. (2021)]. Interestingly, lifestyle factors, diet in particular, have a significant impact on its abundance. In non-Westernized populations with diets rich of carbohydrates and fibers, the *P. copri* complex is a common inhabitant of the gut, while different bacterial taxa dominate in populations with Western-type diets. On one hand, the *P. copri* complex seems to possess beneficial metabolic effects on human health but, on the other hand, potential detrimental effects may also exist (Ley, 2016; Tett et al., 2021). The impact of diet on oral *Prevotella* species is not known.

### *Prevotella* Involvement in Gastrointestinal Diseases

In the human esophagus, gastroesophageal reflux disease is a rather common pathological condition, where the relaxation of the sphincter muscle at the lower esophagus allows acidic stomach contents frequently flow back into the esophagus and further into the pharynx^1^. This constant leakage irritates the epithelial surface, causing reflux symptoms or esophagitis, which may lead to Barrett’s esophagus with a disturbed epithelium structure. The potential role of changes in the esophageal and/or salivary microbiota has been studied and, in this context, the genus *Prevotella* is of interest (Liu et al., 2013; Kawar et al., 2021). Bacterial communities of the distal esophagus were examined at the phylum and genus levels in biopsy samples collected from Japanese patients with either normal esophagus, reflux esophagitis, or Barrett’s esophagus (Liu et al., 2013). The study revealed Bacteroidetes among the major phyla, and the proportions of *Prevotella* clones in the samples were 3, 5, and 12%, respectively. Of the six patients in each group, the number of *Prevotella*-positive patients was three, four, and six, respectively. A distinct bacterial composition seen between the groups was assumed to indicate an association with an esophageal health status and disease type (Liu et al., 2013). Recently, Kawar et al. (2021) compared the bacterial composition of salivary samples collected from reflux patients treated with proton pump inhibitors to that from non-medicated reflux patients and healthy controls. Abundant taxa present in the samples from the latter two groups differed considerably, including reduced abundances of *P. melaninogenica* and *P. pallens* in saliva of reflux patients. It was assumed that a lowered pH in the oral cavity of non-medicated patients explains this difference, since no significant difference was found between saliva of medicated reflux patients and healthy controls (Kawar et al., 2021).

Bacterial compositions of the stomach and duodenum were examined in 98 Korean patients with symptomatic gastritis (Han et al., 2019). It was demonstrated that the gastric and duodenal microbiota differ from each other. Symptom scores only weakly correlated with abundance of gastric *H. pylori* but, instead, correlated more strongly with the duodenal microbiota. An interesting gender-related finding was the higher relative abundance of *P. pallens* in the stomach of women than in that of men. There was a negative correlation with relative abundance of *P. pallens* and the severity of symptoms in the stomach. Positive correlations were found with *P. nanceiensis* and *A. rava* in the duodenum. According to the authors, other factors than *H. pylori* need to be taken into account in symptomatic gastritis (Han et al., 2019).

A novel *Prevotella* species was detected for the first time in a biopsy taken from the jejunum of a child with celiac disease and was named as *P. jejuni* due to its localization in this part of the small intestine (Hedberg et al., 2013). However, this species seems to be a common inhabitant of the oral cavity and especially in saliva, similarly to its close relative, *P. melaninogenica* (own unpublished data). Alterations in the salivary microbiota and metabolome in celiac children, who had been under glutein-free diet at least for 2 years, were examined and compared to those of healthy control children (Francavilla et al., 2014). The number of total cultivable anaerobes differed between the groups, with reduced amounts found in children with celiac disease. Members of the phylum Bacteroidetes, among those *P. nanceiensis*, dominated in saliva, with increased abundance in celiac children. It was concluded that the diet change of 2 years may not be enough to restore the salivary microbiota (Francavilla et al., 2014).

Although the relation of the oral microbiota to inflammatory bowel diseases (IBD) is still controversial, there is evidence on their connection to dysbiotic bacterial communities in saliva (Said et al., 2014; Xun et al., 2018; Qi et al., 2021). Dysbiosis with an increased relative abundance of Bacteroidetes, in turn, causes an elevated inflammatory response, which is also seen in saliva as increased levels of cytokines like interleukin-1β (Said et al., 2014). No alterations were observed in the richness and diversity of bacterial communities between saliva from IBD patients and healthy controls, whereas the bacterial composition in saliva varied (Said et al., 2014; Qi et al., 2021). While abundance of *Streptococcus* decreased, that of *Prevotella* (besides *Veillonella*) and *P. melaninogenica* significantly increased in both Crohn’s disease and ulcerative colitis patients. The genus *Prevotella* was suggested as a potential indicator related to Crohn’s disease (Qi et al., 2021). Moreover, the dysbiotic salivary composition contributed to aggravated immune disorders in IBD patients.

### *Prevotella* in Cancers of the Digestive Tract Above the Waistline

In the esophagus, cancer types differ depending on the geographical location; in the East, squamous cell carcinoma (SCC) dominates, whereas adenocarcinoma is more common in Western countries (Sung et al., 2021). In the first study to examine bacterial infection as a background factor for esophageal SCC and to identify bacteria that could predict the cancer prognosis, the genus *Prevotella* came up as a potential prognostic indicator for this cancer type (Liu et al., 2018). Patients with lymph node metastasis had higher abundance of *Prevotella* and *Treponema* than patients without metastatic findings, while increased abundance of *Prevotella* combined with *Streptococcus* affected survival rates, predicting poor prognosis. Alterations of the esophageal microbiota have also been studied in connection to Barrett’s esophagus and esophageal adenocarcinoma (Lopetuso et al., 2020). The former condition with changes in the epithelial structure with acid stress and inflammation is considered to expose to metaplastic mucosa. Again, abundance of *Prevotella* was significantly increased, however, species-level shifts showed decreased proportions of *P. melaninogenica* in samples from Barrett’s esophageal mucosa with or without cancer but increased proportions of unclassified *Prevotella*. *P. histicola* was common on mucosae of Barrett’s esophagus, while *P. nigrescens* proportions on metaplastic mucosa were slightly increased (Lopetuso et al., 2020). The authors reported a co-exclusion association between *Streptococcus* and *Prevotella*; a significant reduction in relative abundance of *Streptococcus* and corresponding increase in abundance of *Prevotella* were observed on mucosae of both Barrett’s esophagus and adenocarcinoma.

A decade ago, bacterial communities of the stomach in gastric cancer patients were described for the first time by molecular techniques and compared to those in dyspeptic patients with normal mucosa as controls (Dicksved et al., 2009). The second most dominant phylum proved to be Bacteroidetes, composing mainly of *Prevotella* taxa; among known species, *Prevotella multiformis*, *P. nigrescens*, *P. oris*, and *A. tannerae* were recognized in gastric cancer patients. It can be speculated whether changed conditions due to an increased use of acid-reducing drugs or neoplastic mucosa would allow the colonization of atypical bacteria in the stomach and progression of cancer (Dicksved et al., 2009; Coker et al., 2018). Coker et al. (2018) investigated mucosal biopsy specimens collected from superficial gastritis, atrophic gastritis, intestinal metaplasia, and gastric cancer patients, and detected highest abundance of several oral bacteria, among those *P. intermedia* and *P. oris*, in gastric cancer samples. It was emphasized, however, that it remains to be elucidated in targeted studies whether bacteria enriched are passengers or drivers of carcinogenesis (Coker et al., 2018).

Poor oral hygiene is suggested as a moderate risk factor for pancreatic cancer (Huang et al., 2016). This may be connected to increased amounts of microbial biofilms on oral surfaces and activation of host inflammatory response. In a recent study, a large variety of samples from the gastrointestinal tract, including oral (saliva and swabs from tongue, buccal mucosa, and gingiva), upper intestinal (duodenum tissue and swabs from jejunum and bile duct), and pancreatic (tumor or normal tissue and pancreatic duct) samples were examined to clarify the microbiota in patients with pancreatic cancer or other diseases of the pancreas or the foregut (Chung et al., 2021). *Streptococcus*, *Veillonella*, and *Prevotella* were the most shared genera between oral and gut or pancreatic samples, and *P. veroralis* among the most frequently shared species. Amplicon Sequence Variants had some overlaps between the close sites within the mouth and within the pancreas. In co-abundance analyses, distinct strain-level cluster patterns were observed among microbial findings in buccal swabs, saliva, duodenal tissue, jejunal swabs, and pancreatic tumor tissue. In the latter sample site, *P. nigrescens* was found among dominating species in one cluster (Chung et al., 2021).

## *Prevotella* in Bacterial Communities of the Respiratory Tract

### *Prevotella* as Members of the Core Microbiota in the Respiratory Tract

A gradual maturation of the early microbiota of the lower respiratory tract occurs within less than 2 months after birth in full-term infants (Pattaroni et al., 2018). Three distinct colonization patterns were recognized in tracheal aspirates and were explained by distinct microenvironments in preterm and term infants. Of those, a mixed microbial profile consisted of a balanced composition of six genera, including *Streptococcus* and *Neisseria* as keystone genera, and anaerobic *Prevotella*, *Veillonella*, *Porphyromonas*, and *Fusobacterium*. This combination stayed stable across the first year of life (Pattaroni et al., 2018). Interestingly, this is a typical bacterial composition for the early microbiota established in the mouth during the first year of life (Könönen et al., 1999; Könönen, 2005). It also resembles the composition of the lung microbiota of adults during health (Bassis et al., 2015).

Similar interacting consortia as seen in the oral cavity (but not in the nasal cavity) can be observed in the lower respiratory tract, even though relative abundance and diversity richness are lower in the lungs (Bassis et al., 2015). Indeed, the oropharynx is considered the principal origin for the lung bacteriome during health (Bassis et al., 2015; Dickson et al., 2016). At the genus level, *Prevotella* is consistently among the core bacterial communities of the respiratory tract (Bassis et al., 2015; Einarsson et al., 2019). However, methodologies assessing the bacterial taxa at the species level, and even at the strain level, are necessary for discovering their source and role as beneficial, harmless or potentially harmful members of the bacterial communities at a specific body site. Noteworthy is that the *Prevotella* genus accommodates a high number of species with distinct clinical significance.

Valuable, detailed species-level oropharyngeal data assessed by in-depth sequencing are available from a study where tonsillar crypts in 2- to 4-year-old children and young adults were examined during recurrent tonsillitis and were compared to tonsillar crypts in children with tonsillar hyperplasia but without inflammation and those in healthy adult controls (Jensen et al., 2013). *Streptococcus* and *Prevotella* were found in all 20 samples from children and high *Prevotella* abundance was observed. Recurrent tonsillitis associated with a shift in the bacterial composition, especially with increased *P. melaninogenica/P. histicola* in adults. Typical oral *Prevotella* species, including *Prevotella buccae*, *P. dentalis*, *P. denticola*, *Prevotella fusca*, *Prevotella micans*, *P. oralis*, *P. oris*, *P. pallens*, *Prevotella salivae*, and *P. veroralis*, were more abundant in adults but *Prevotella saccharolytica* in children. *P. intermedia* was absent, except for a healthy adult with a high quantity. The recoveries of *P. nanceiensis* and *P. pleuritidis* from the oropharynx give support for their oral habitat. These study results indicate that a core microbiome, with a few significant genera, is present in tonsillar crypts regardless of individuals’ age and health status (Jensen et al., 2013).

Recently, a potential link between the microbial community composition and lung homeostasis was examined by analyzing bronchoalveolar lavage samples from a longitudinally followed post-transplant study population (Das et al., 2021). Although the lung microbiota proved to be highly variable, there were a few bacterial taxa with high prevalence and/or abundance, and the majority of them were either obligate or facultative anaerobes. Among the most prevalent species were *P. melaninogenica*, *Veillonella atypica*, *Veillonella dispar*, *Streptococcus mitis*, and *Granulicatella adiacens* (all typical recoveries from the oral cavity). The microbiota was categorized into four distinct compositional states (pneumotypes) where the balanced pneumotype represented a diverse bacterial community, resembling that in the oropharynx and including *P. melaninogenica*, which occurred in 97.4% of the samples (Das et al., 2021). In addition, this pneumotype had a high immune-modulatory activity and preserved lung stability.

Although oral bacteria get access to proximal airways via microaspiration, their growth at high densities is prevented by the continuous mucociliary clearance (Bassis et al., 2015). A recent study using a mouse model (Wu et al., 2021) demonstrated that the episodic aspiration of oral commensals, such as *P. melaninogenica*, *Veillonella parvula*, and *S. mitis*, leads to dysbiosis and low-dose inflammation in the lower airways; the consequence is a reduced susceptibility to pathogenic *Streptococcus pneumoniae* via activation of pulmonary Th17 cells. The shift in the human lung microbiome from the phylum Bacteroidetes in health to Gammaproteobacteria in disease indicates that *Prevotella*-activated Th17 response is an essential part of the pathogen recognition and suppression system of a healthy lung environment (Huffnagle et al., 2017). Anaerobic bacteria, especially *Prevotella*, are frequent recoveries from clinical respiratory specimens; however, understanding of their contribution to lung diseases is not clear yet.

### *Prevotella* Involvement in Acute Diseases of the Respiratory Tract

Along with high research interest targeted to the COVID-19 pandemic, an increasing number of reports on the potential involvement of oral bacteria in the disease persistence and treatment outcome are available in the current literature. Interestingly, also *Prevotella* organisms, being analyzed from salivary, oropharyngeal, and bronchoalveolar lavage samples examined for SARS-CoV-2, have come up recently in this context. The microbiome and SARS-CoV-2 viral loads in saliva were compared between hospitalized COVID-19 and control patients (Miller et al., 2021). Although no significant difference in their bacterial compositions was found, the abundance of some taxa associated with the viral load in saliva; here, of special interest is enriched *P. pallens* but reduced *P. denticola* and *P. oris* in saliva of COVID-19 patients (Miller et al., 2021). Xiong et al. (2021) examined the difference in the microbial composition between SARS-CoV-2-positive and -negative pharyngeal swab samples collected from symptomatic patients with cough and fever. A significantly reduced species richness was seen in COVID-19 samples. The top-3 genera enriched were *Streptococcus, Prevotella*, and *Campylobacter*. Changes in abundance were seen also for several *Prevotella* species, such as *P. denticola*, *P. oris*, *P. jejuni*, *P. intermedia*, *P. melaninogenica*, *P. fusca*, and *P. scopos*, which were among the 37 species distinctive for the healthy and diseased individuals, and most of them separated the symptomatic COVID-19 and non-COVID groups from each other (Xiong et al., 2021). A dysbiotic oropharyngeal microbiota with gram-negative commensals, considered pathobionts due to their lipopolysaccharide production, has been connected to a so-called long-COVID disease (Haran et al., 2021). Among 164 patients with various types of symptoms, increased abundances of several *Prevotella* species were among the top predicting taxa from swabs of the posterior oropharynx: *P. denticola*, *P. nigrescens*, *P. histicola*, and *P. oulorum* in the patient group with ongoing symptoms, and *P. denticola*, *P. melaninogenica*, *P. jejuni*, and *P. nigrescens* in the determined long-COVID group (Haran et al., 2021). Sulaiman et al. (2021) examined a hospitalized cohort of 589 critically ill COVID-19 patients, characterizing their lung microbiome from bronchoalveolar lavage samples. Especially interesting were the findings of two oral commensals, *P. oris* and *Mycoplasma salivarium*, among the most dominant, functionally active microbial taxa. While *M. salivarium* was enriched in the deceased and >28-day mechanically ventilated groups, *P. oris* was enriched in the ≤28-day group. It was suggested that dissimilar microbial pressures related to host factors could explain the difference between the groups (Sulaiman et al., 2021). These studies indicate that the microbiome of the host plays a role in COVID-19 outcome.

Oral bacteria present in saliva can promote aspiration pneumonia via colonizing on mucosal surfaces, affecting immune response of epithelial cells, and producing proinflammatory cytokines and degradative enzymes, but dispersal via hematogenous route is also an option (Scannapieco, 2021). Older age and supine position as well as poor oral hygiene increase the risk for aspiration of bacteria from the oral cavity to lower parts of the respiratory tract (Hasegawa et al., 2014; Scannapieco, 2021). In pneumonias as well as pleural empyema, both anaerobes and oral bacteria, which can be missed by conventional culture, are more frequent findings by molecular methods (Yamasaki et al., 2013; Dyrhovden et al., 2019; Aoki et al., 2021). In community-acquired pneumonia, approximately 8% of bronchoalveolar lavage specimens from 64 hospitalized pneumonia patients were positive for *Prevotella/Alloprevotella*, three with *P. veroralis*, and *P. melaninogenica* and *A. tannerae* one each (Yamasaki et al., 2013). These findings came from mild cases, however, their pathogenic role remained unknown. Attempts to recover bacterial taxa regardless of their expected pathogenicity or quantity from respiratory specimens revealed *Prevotella* species in the majority of 17 aspiration pneumonias and eight lung abscesses (Aoki et al., 2021). Only occasional *Prevotella* recoveries, including *P. buccae*, *P. oris*, *P. pleuritidis*, and *A. tannerae*, came from 27 pleural empyema with poorly described etiology but potentially of oral origin (Dyrhovden et al., 2019).

### *Prevotella* Involvement in Chronic Diseases of the Respiratory Tract

Chronic diseases of the airways are characterized by a reduced capability of eliminating microbes (Dickson et al., 2016). This may allow a persistent colonization of opportunistic pathogens, such as *Pseudomonas aeruginosa* or *Haemophilus influenzae*, with harmful consequences for respiratory health. During exacerbation, there are acute periods resulting in both local and systemic inflammation and worsened lung function. Due to culture-independent methods and increased research interest in the role of anaerobic bacteria and their function, it has been shown that dysbiotic bacterial compositions are, indeed, involved in inflammation of the respiratory airways (Dickson et al., 2016; Huffnagle et al., 2017). The presence of *Prevotella* in the lower respiratory tract is related to the Th17 activation and differentiation, as defined by Th17-chemoattractant chemokine concentrations and STAT3 expression, respectively (Segal et al., 2016). However, the role of *Prevotella*-mediated Th17 activation in the maintenance of lung health is not fully elucidated yet. A recent mouse-model study demonstrated that the episodic aspiration of oral commensals (*P. melaninogenica*, *V. parvula*, and *S. mitis*) leads to dysbiosis and low-dose inflammation in lower airways, decreasing the susceptibility to the respiratory pathogen *S. pneumoniae* via activation of pulmonary Th17 cells (Wu et al., 2021).

Reduced abundances of the phylum Bacteroidetes and the genus *Prevotella* have been observed in the oropharynx of patients with asthma and chronic obstructive pulmonary disease (COPD) as well as in bronchial washings in COPD patients (Hilty et al., 2010; Park et al., 2014; Einarsson et al., 2016). A recent study examined pediatric asthma-associated alterations in the respiratory microbiota connected to host metabolism and responses, showing that several *Prevotella* species were enriched in the control group as well as *P. pallens* and *Prevotella* oral taxon 306 having an inverse correlation with total and allergen-specific IgE levels (Chiu et al., 2020). In the oropharynx of 13 adult asthma patients, *Prevotella* proved to be the most abundant genus and *P. melaninogenica*, *P. pallens*, and *P. nigrescens*, in descending order, most abundant *Prevotella* species (Lopes Dos Santos Santiago et al., 2017). Although *P. melaninogenica* and *S. mitis/S. pneumonia*e were the most abundant species, the bacterial compositions did not differ from those found in non-asthmatic individuals. As regards COPD, a recent study characterizing a potential association between the microbiota and risk for exacerbation or airflow limitation revealed significantly reduced proportions of *P. histicola*, *Gemella morbillorum*, and *Streptococcus gordonii* in sputum of patients with high risk of COPD exacerbation (Yang et al., 2021). The authors assumed that this kind of alteration in the resident microbiota to a dysbiotic direction could enhance inflammation in respiratory mucosae. In a mouse model, *P. melaninogenica*, *P. nanceiensis*, and *P. salivae* strains were shown to induce COPD-like symptoms via activation of neutrophils and elevating cytokine expression in a TLR-2 dependent manner (Larsen et al., 2014). An interesting finding was that only whole cells but not lipopolysaccharide of *Prevotella* initiated these symptoms, indicating that TLR-4 does not take part in the cellular response against *Prevotella* seen in healthy airways of humans. In a previous study, the authors demonstrated that the same *Prevotella* strains were able to reduce the expression of *Haemophilus*-induced human dendritic cell IL-12p70, but not IL-23 and IL-10 expressions (Larsen et al., 2012). Later, it was demonstrated that *Prevotella* outer membrane proteins (OMPs) are responsible for the Th17 development, activation, and IL-17B and IL-17A expression, which eventually promote pulmonary fibrosis (Yang et al., 2019). Noteworthy is that OMPs of *Prevotella* contain lipopolysaccharides and lipoproteins, which stimulate IL-17 expression via the TLR-Myd88 signaling pathway (Yang et al., 2019). These results indicate that commensal *Prevotella* organisms not only contribute to the aggravation of airway immune response, but also enable the regulation of pathogen-induced immune response.

Also in other chronic diseases of the lower respiratory tract, like bronchiectasis and cystic fibrosis, *Prevotella* is among the predominant findings (Tunney et al., 2013; Renwick et al., 2014; Muhlebach et al., 2018; Einarsson et al., 2019). For example, a multi-center study, including over 200 participants with age ranging from childhood to mid-adulthood and with different genetic and geographic backgrounds, examined whether there is a link between strict anaerobes and the severity of cystic fibrosis (Muhlebach et al., 2018). Across all ages, *Streptococcus* and *Prevotella* had the highest detection rates in sputum samples, 82 and 51%, respectively. Contrasting to the high prevalence of *Prevotella*, its abundance appeared to be low. Interestingly, the presence of anaerobes associated with phenotypically milder disease, whereas *Pseudomonas* (*P. aeruginosa*), the typical pathogen in cystic fibrosis, associated with severe disease (Muhlebach et al., 2018). Recently, Einarsson et al. (2019) assessed microbial community structures within the airways and clarified how various taxa are distributed in communities representing health or chronic disease (here: bronchiectasis and cystic fibrosis). The “core” community was composed of members of the genera *Streptococcus*, *Veillonella*, *Prevotella*, *Granulicatella*, and *Fusobacterium*, while skewed community structures were found in cystic fibrosis and bronchiectasis samples. Notably, anaerobic bacteria, e.g., *Prevotella* and *Veillonella*, proved to affect the variance within the airways, interacting with opportunistic lower airway pathogens (Einarsson et al., 2019).

Some species-level data are available on *Prevotella* in cystic fibrosis. A recent multi-center study (O’Connor et al., 2021), looking for bacterial communities in bronchoalveolar lavage fluid of 63 diseased and 128 control individuals from infancy to young adulthood, demonstrated the *S. mitis* group (52%) and *P. melaninogenica* (44%) as being the most prevalent bacterial taxa. However, distinct abundance patterns were recognized between the study groups; while the abundance of the *S. mitis* group was high regardless of age in controls, *Staphylococcus aureus* dominated in cystic fibrosis. Low abundance of *P. histicola* was found in part of diseased and control individuals across the age spectrum, and interestingly, *P. oris* was detected at high abundance in some diseased individuals (O’Connor et al., 2021). Based on the comparison of bronchoalveolar fluid and oropharyngeal swab samples, overall diversity of the upper and lower airway microbiome is similar in clinically stable children with cystic fibrosis (Renwick et al., 2014). However, bacterial communities in lower airways significantly differed between cystic fibrosis and control children; while *P. veroralis* was absent in cystic fibrosis, it was common in controls. Pulsed-field gel electrophoresis patterns were produced in a study targeting to reveal the degree of clonal similarity of 42 *Prevotella* isolates collected from sputum samples during stable, exacerbated, and post-exacerbation periods (Gilpin et al., 2017). Initial sampling was performed during clinical stability, and the *Prevotella* findings included *P. denticola*, *P. histicola*, *P. melaninogenica*, *P. nigrescens*, and *P. salivae*. Seven isolates could not be definitely identified but remained as *P. veroralis/P. histicola* or *P. melaninogenica/P. histicola*. Genotyping analysis allowed recognizing similar banding patterns (genotypes) during the follow-up. It was suggested that, instead of repeated acquisition, a persistent colonization of *Prevotella* species had occurred in patients with cystic fibrosis (Gilpin et al., 2017). In an experimental study, cystic fibrosis bronchial epithelial cells were exposed to *P. histicola* or *P. nigrescens* (Bertelsen et al., 2021). Both strains were able to induce disrupted NF-κB(p65) and MAPK activations via suppressing TLR-4 and stimulating TLR-2 in epithelial cells. Infection with a *P. nigrescens* strain induced only low levels of p65-mediated inflammation compared to inflammatory response of a *P. aeruginosa* strain from the same patient (Bertelsen et al., 2020). The authors speculated that by TLR-2 signaling, and by reducing TLR-4 release and IL-6 and IL-8 production, *Prevotella* may inhibit the growth of the major pathogen, *P. aeruginosa*, and have a beneficial effect on immune response in the lungs affected by cystic fibrosis.

## Summary

Due to increased research interest in commensal bacteria in humans, there is now considerable evidence on the complex nature of commensal bacterial communities in the lower airways as well as gastrointestinal tract above the waistline, including the esophagus, stomach, and upper part of the small intestine. It is obvious that oral members of the genus *Prevotella* play an important role in health and disease at these body sites. In the gastrointestinal tract, the presence of *Prevotella* may influence, positively or negatively, the severity of disease, such as reflux disease, gastritis, IBD, and different cancer types. In the respiratory tract, current research has brought information on the potential involvement of oral bacteria, including some *Prevotella* organisms, in COVID-19 persistence and treatment outcome. As regards *Prevotella* species in chronic respiratory diseases, the current data report on reduced abundance of anaerobes, especially *Prevotella*, which indicates a disruption of homeostatic respiratory microbiota, potentially exposing to lung disease progression. To date, there is only a limited number of mechanistic studies to explain the relation between specific *Prevotella* species involved in diseases in the respiratory and gastrointestinal tracts. The wide intra-genus variation and distinct properties of individual species within the genus *Prevotella* call for further studies on oral *Prevotella* species and their involvement inside and outside the oral cavity to clarify their impact on human health and disease.

## Author Contributions

EK conceptualized the manuscript and was responsible for the content dealing with microbes. UKG was responsible for immune-inflammatory aspects. Both authors read and approved the final manuscript.

## Conflict of Interest

The authors declare that the research was conducted in the absence of any commercial or financial relationships that could be construed as a potential conflict of interest.

## Publisher’s Note

All claims expressed in this article are solely those of the authors and do not necessarily represent those of their affiliated organizations, or those of the publisher, the editors and the reviewers. Any product that may be evaluated in this article, or claim that may be made by its manufacturer, is not guaranteed or endorsed by the publisher.

## Footnotes

1
https://www.hopkinsmedicine.org/gastroenterology_hepatology/_pdfs/esophagus_stomach/gastroesophageal_reflux_disease.pdf
